# Supplementary material for: The POLR3G Subunit of Human RNA Polymerase III Regulates Tumorigenesis and Metastasis in Triple-Negative Breast Cancer
Source: Cancers (Basel). 2022 Nov 22;14(23):5732. doi: 10.3390/cancers14235732 (PMC9735567; doi:10.3390/cancers14235732)
Supplement: Supplementary file 1 [file cancers-14-05732-s001.zip › cancers-2022480-supplementary.pdf]

Supplementary Materials: The POLR3G Subunit of Human RNA Polymerase III Regulates Tumorigenesis and Metastasis in Triple-Negative Breast Cancer

Wiebke Lautré, Elodie Richard, Jean-Paul Feugeas, Hélène Dumay-Odelot and Martin Teichmann

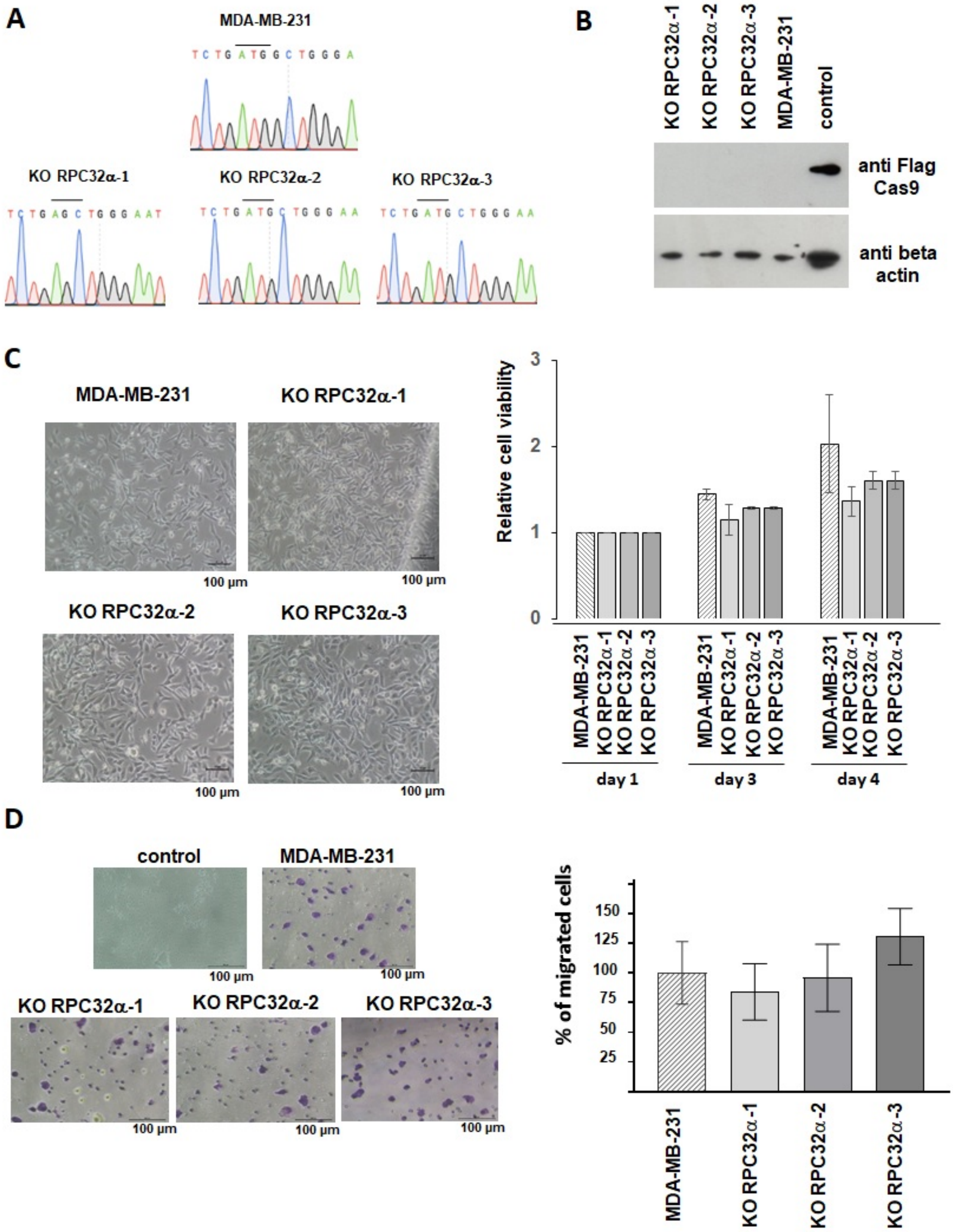

Figure S1. Identification of POLR3G KOs (3GKOs) and analysis of their 2D growth and migratory capacities compared to the MDA-MB-231 cell line. (A) Identification of 3GKO cell lines by Sanger sequencing. (B) Western blot analysis of Cas9 expression in MDA-MB231 and 3GKO cells, as well as in Cas9-transfected MDA-MB231 cells (control). Analyzed cell lines are appropriately depicted

above the ECL autoradiograph. (C) Representative images of MDA-MB231 cells and of three POLR3G KO clones grown on Petri dishes are shown to the left. Cell proliferation as indicator of viability was determined by MTT assays according to the manufacturer's protocol (CellTiterAqQueous One Solution Cell Proliferation assay, Promega). A graphic representation of mean proliferation ± SEM of RPC32α knockout (3GKO) cell lines compared to MDA-MB-231 cells is shown to the right. (D) 3GKO clones showed the same migratory behavior as the MDA-MB231 mother cell line. Representative microscope images are displayed to the left and graphic presentation of the quantifications of migration in Transwell assays without matrigel are shown to the right. Scale bar: 100 µm. Data are presented as the mean of three independent experiments ± SEM.

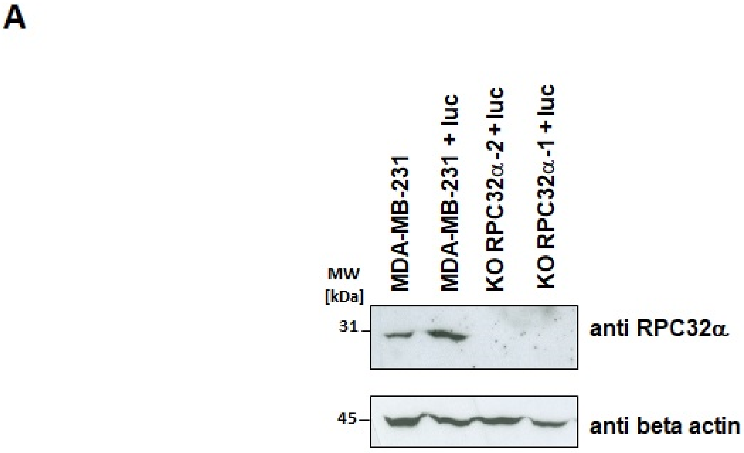

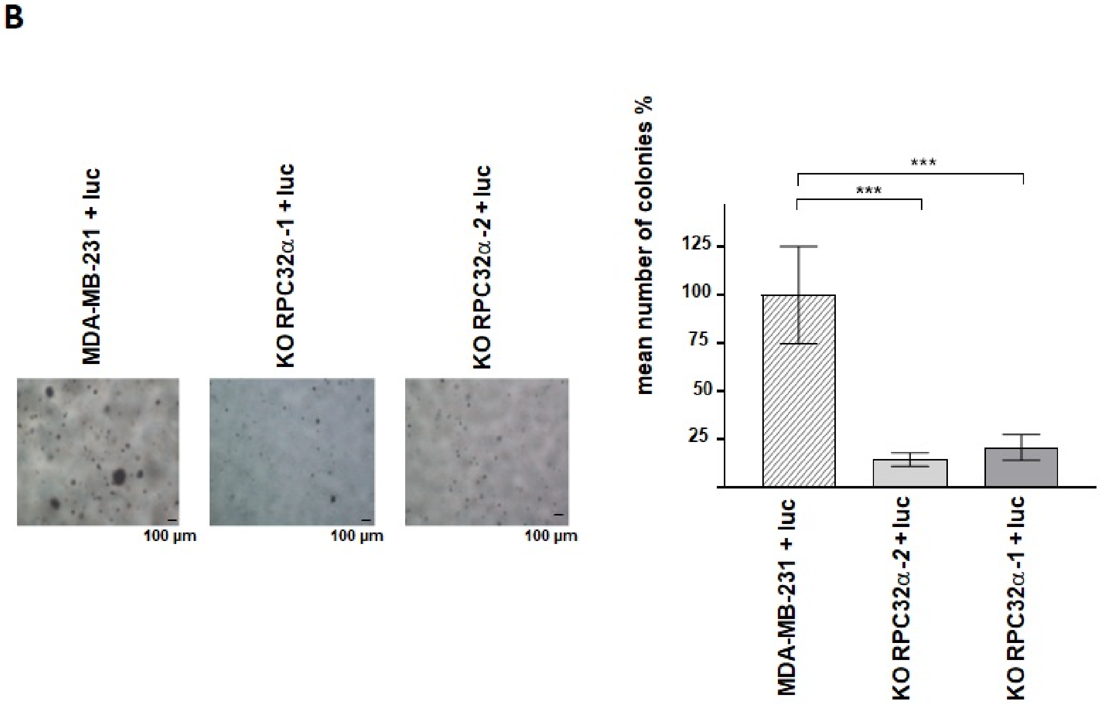

Figure S2. The addition of the luciferase gene in the different cell lines does not affect POLR3G expression and colony formation in vitro. (A) Western blot analysis of RPC32α expression. + luc represents the addition of the luciferase gene in the cell line. (B) Soft agar assays. Representative microscope images of colony formation stained by crystal violet are shown to the left. Graphs shown to the right represent relative colony numbers in MDA-MB-231 and RPC32α knockout cell lines expressing the luciferase gene. Scale bar: 100 µm. Data are presented as the mean of three independent experiments ± SEM (****p* < 0.001 compared with MDA-MB-231 containing luciferase gene).

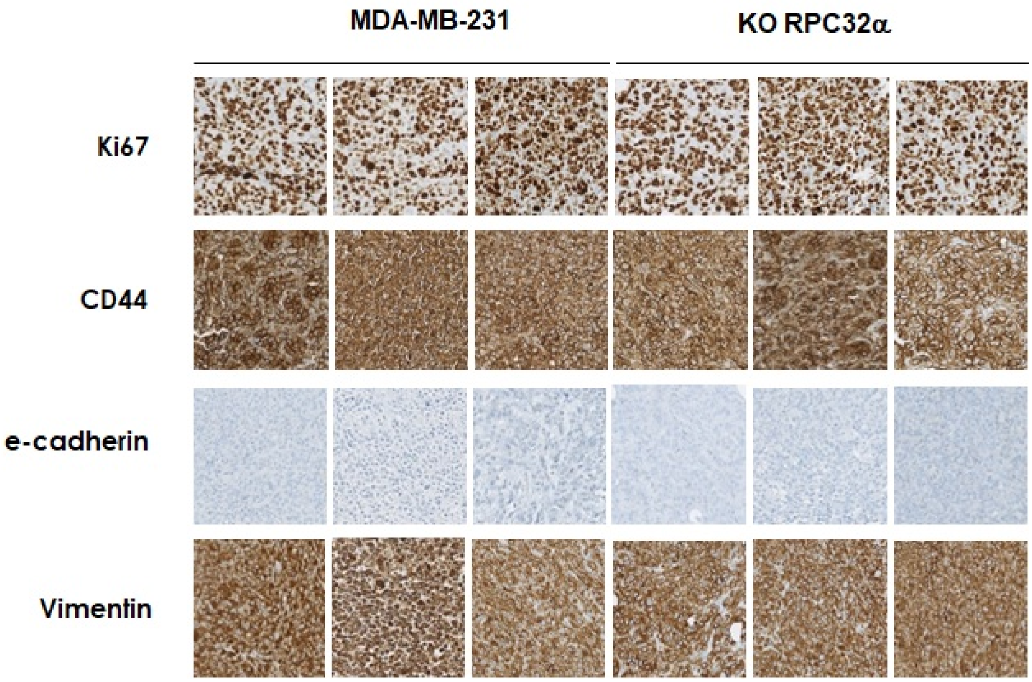

Figure S3. Immunohistochemical analyses of tumors formed by MDA-MB-231 or RPC32α KO (3GKO) cells. Ki67, CD44, e-cadherin and vimentin were detected by antibodies as described in Materials and methods. Squares in the three lanes to the left show representative samples derived from tumors formed by MDA-MB-231 cells, those in the three lanes to the right are derived from tumors formed by 3GKO cells. Stained proteins are indicated to the left of the rows.

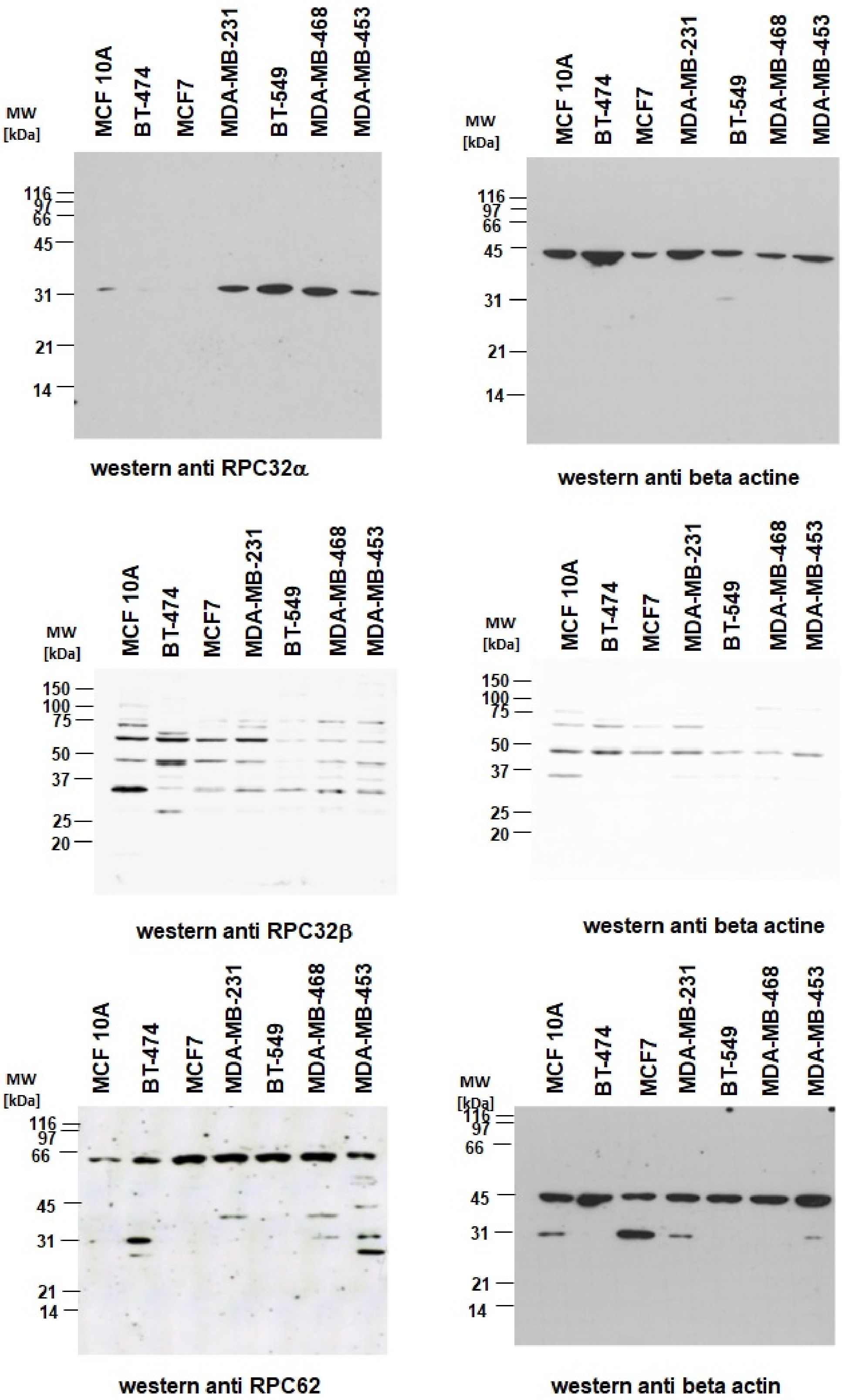

Figure S4. Uncropped images of Western Blots shown in Figures 3B and C.

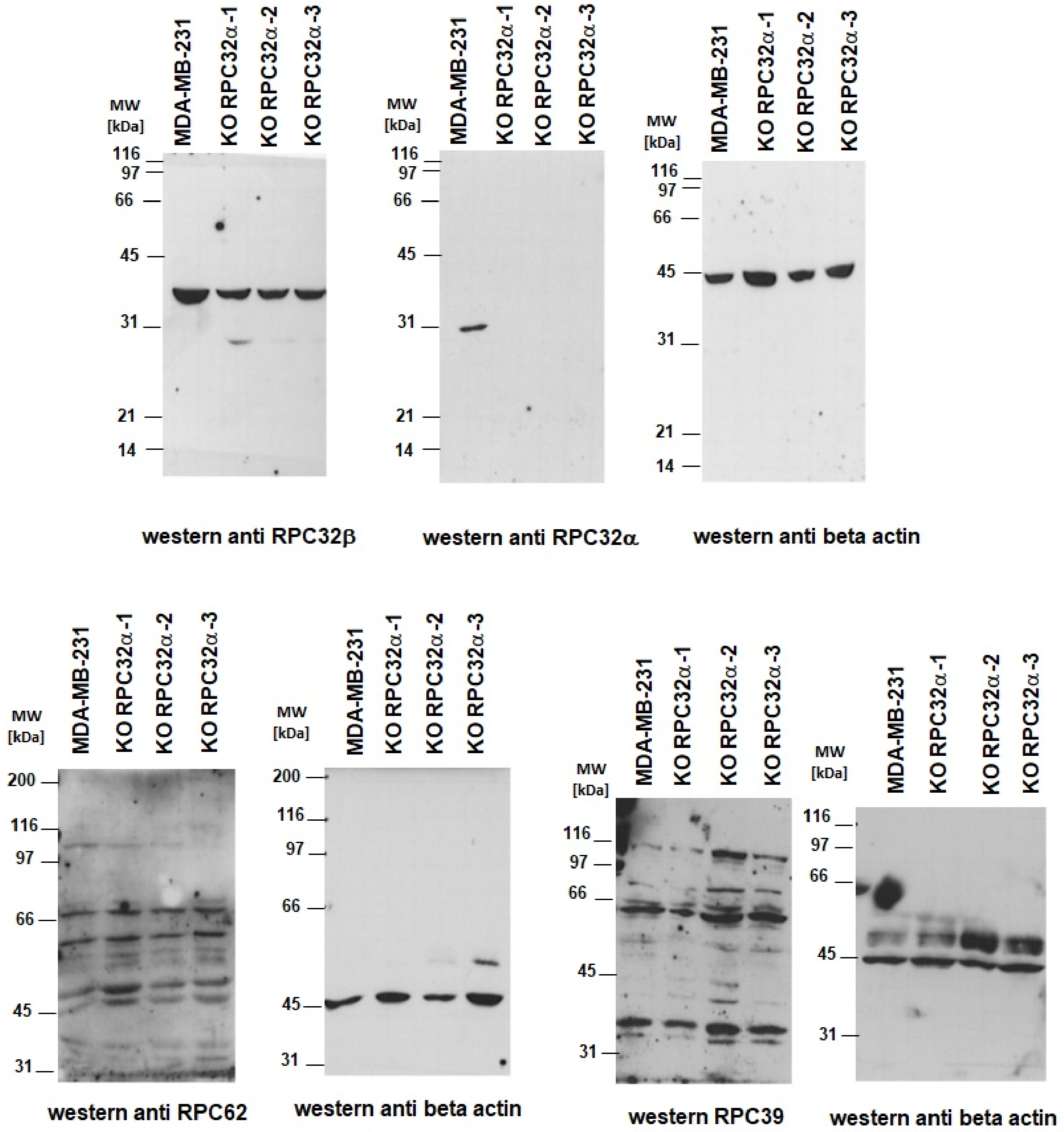

Figure S5. Uncropped images of Western Blots shown in Figures 4A and B.

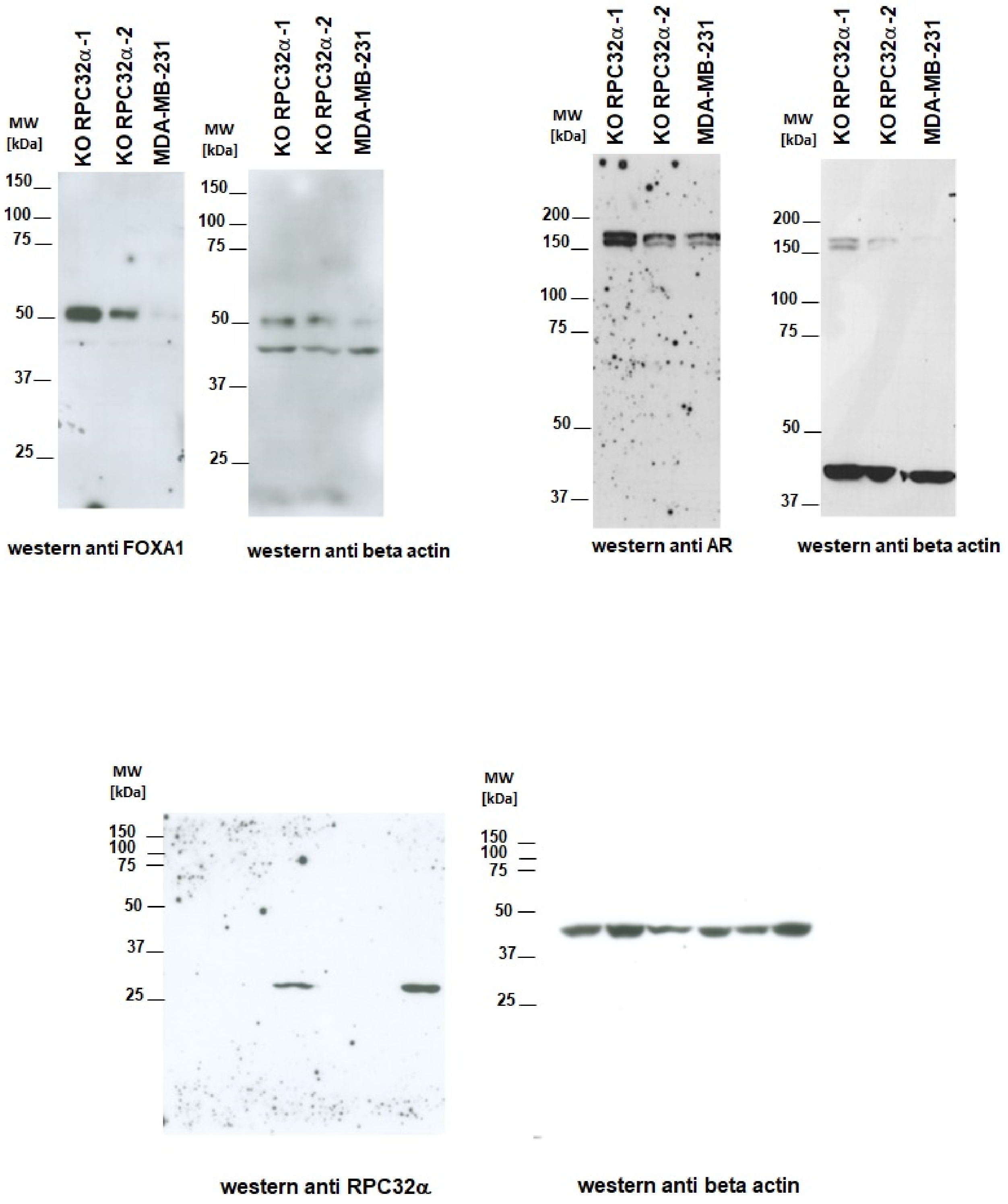

Figure S6. Uncropped images of Western Blots shown in Figures 8B and C.

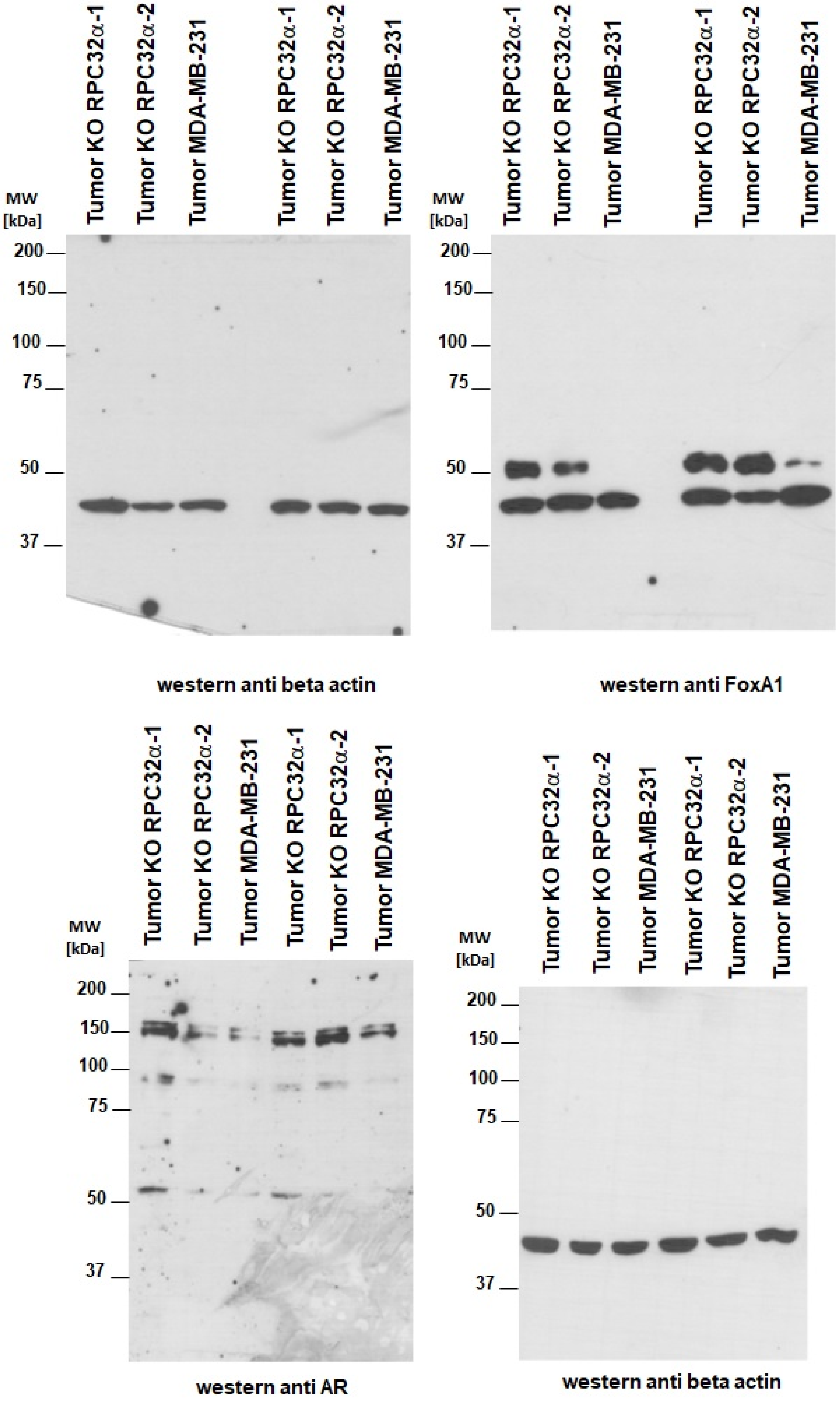

Figure S7. Uncropped images of Western Blots shown in Figures 8D.

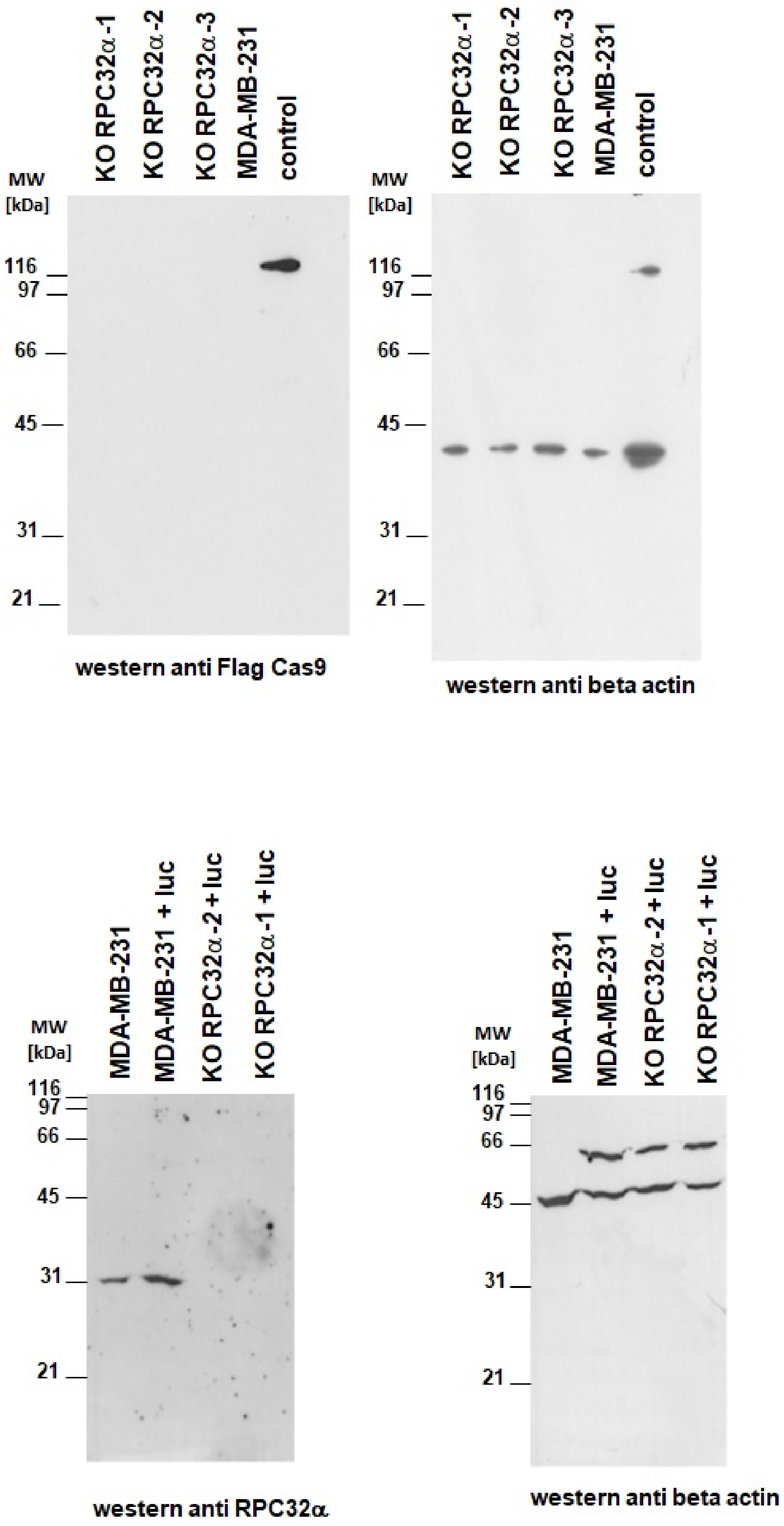

Figure S8. Uncropped images of Western Blots shown in Figures S1B (upper panel) and S2A (lower panel).

Table S1. Expression analysis of Pol III transcription components in different breast cancer types and normal tissue. Transcriptome analysis was performed using 2627 breast samples as described in Materials and Methods. Breast cancer types were assigned by PAM50 method (genefu R package) as luminal A, luminal B, HER2 or Basal. Differential gene expression analysis was carried out according to the general linear model (glm R package). Log2 values are shown. FC basal vs Lum: Fold change between basal and luminal groups. Genes are ordered according to their fold change (difference between log means). FDR: False discovery, p.adjust R package rate.

Gene	*p*-Value	FDR	FC Basal vs Lum	Basal	Her2	Luminal	Normal Breast
POLR3G	3×10^{-108}	1×10^{-106}	1.7	4.43	2.81	2.77	3.41
POLR2F	5×10^{-79}	9×10^{-78}	0.9	9.75	9.06	8.87	8.79
POLR1C	8×10^{-50}	7×10^{-49}	0.8	7.44	6.83	6.60	6.08
GTF3C6	2×10^{-61}	2×10^{-60}	0.6	11.95	11.70	11.33	10.99
POLR3C	7×10^{-35}	3×10^{-34}	0.6	6.61	5.89	6.02	6.12
POLR2D	2×10^{-47}	2×10^{-46}	0.6	7.19	7.10	6.63	6.62
GTF3C5	8×10^{-40}	5×10^{-39}	0.5	8.82	8.62	8.29	8.42
POLR3D	4×10^{-47}	2×10^{-46}	0.5	6.34	5.90	5.81	5.97
GTF3C4	1×10^{-30}	5×10^{-30}	0.5	6.71	6.37	6.19	5.06
SNAPC1	5×10^{-20}	1×10^{-19}	0.5	7.29	7.16	6.80	6.92
GTF3C2	7×10^{-25}	2×10^{-24}	0.5	4.70	4.54	4.21	3.39
SNAPC3	6×10^{-23}	2×10^{-22}	0.5	8.43	7.69	7.98	7.37
GTF3C3	3×10^{-27}	9×10^{-27}	0.4	8.54	8.26	8.09	8.36
MAF1	1×10^{-07}	2×10^{-07}	0.3	8.90	8.75	8.65	8.50
POLR3A	2×10^{-05}	2×10^{-05}	0.2	5.90	5.81	5.67	6.32
POLR3H	1×10^{-08}	2×10^{-08}	0.2	7.27	7.35	7.04	6.97
TBP	3×10^{-11}	5×10^{-11}	0.2	7.29	7.02	7.08	7.78
POLR3B	9×10^{-02}	1×10^{-01}	0.2	6.85	6.71	6.68	8.14
POLR2K	4×10^{-02}	5×10^{-02}	0.2	9.24	9.24	9.07	9.31
SNAPC4	3×10^{-03}	4×10^{-03}	0.1	5.98	6.12	5.87	5.12
SUB1	2×10^{-04}	2×10^{-04}	0.1	11.41	11.09	11.31	9.10
POLR3F	2×10^{-04}	3×10^{-04}	0.1	7.23	7.00	7.13	7.18
POLR2H	3×10^{-04}	4×10^{-04}	0.1	9.70	9.55	9.61	9.22
SNAPC5	$1 \times 10^{+00}$	$1 \times 10^{+00}$	0.0	7.17	7.29	7.15	7.30
BRF1	2×10^{-01}	2×10^{-01}	0.0	3.03	3.04	3.01	2.84
GTF3A	8×10^{-01}	8×10^{-01}	-0.1	7.98	7.83	8.03	7.11
ZBTB17	2×10^{-03}	3×10^{-03}	-0.1	6.39	6.62	6.47	6.80
POLR1D	3×10^{-01}	3×10^{-01}	-0.1	10.00	10.01	10.10	9.32
SNAPC2	3×10^{-05}	5×10^{-05}	-0.2	4.97	4.97	5.19	4.44
BDP1	2×10^{-07}	3×10^{-07}	-0.3	3.51	3.52	3.76	4.71
BRF2	2×10^{-02}	2×10^{-02}	-0.3	4.95	4.45	5.22	4.60
POLR3GL	1×10^{-16}	2×10^{-16}	-0.3	8.70	8.65	9.01	10.12
POLR2E	3×10^{-06}	5×10^{-06}	-0.3	8.98	9.48	9.31	8.23
POLR3E	2×10^{-18}	4×10^{-18}	-0.4	8.60	8.57	8.95	8.66
POLR2L	2×10^{-25}	7×10^{-25}	-0.6	4.63	4.97	5.19	5.30
GTF3C1	2×10^{-18}	4×10^{-18}	-0.6	7.19	7.74	7.76	6.05
POLR3K	2×10^{-12}	5×10^{-12}	-0.7	7.90	7.93	8.59	6.20

Table S2. Genes with expression patterns correlating to that of POLR3G (across the 2627 samples of this study). The most positively (top) or negatively (bottom) correlated genes are indicated. Gene symbols are depicted in column 1, correlation coefficients with POLR3G in column 2 and correlation coefficients with POLR3GL in column 3.

Gene Symbol	POLR3G	POLR3GL
POLR3G	1.00	-0.03
UGT8	0.59	-0.08
ARL9	0.58	-0.12

TAF4B	0.58	0.00
MLK4	0.56	-0.05
PDSS1	0.56	-0.06
FeRMT1	0.56	-0.09
C21orf91	0.55	-0.06
KLHL7	0.55	0.07
CDCA2	0.54	-0.18
MCAM	0.54	-0.11
SFT2D2	0.54	0.08
ROPN1	0.54	0.10
…..	…..	…..
…..	…..	…..
GATA3	-0.50	-0.01
PRR15	-0.50	0.04
SLC7A8	-0.51	0.06
XBP1	-0.52	0.09
AGR2	-0.53	0.08
SIDT1	-0.53	0.06
AR	-0.54	0.17
SPDeF	-0.57	-0.08
FOXA1	-0.59	0.09
MLPH	-0.61	0.05

Table S3. Oligonucleotide sequences employed in this study.

Name	Target Sequence	
POLR3G	F: 5′-CGCAGGCAAAGGCACAC-3′	R: 5′-CCTCTTTTTCCAATTCCTCCA-3′
POLR3GL	F: 5′-CCAAGAGAGATGTGGAGCGTTATT-3′	R: 5′-TCCAATCGATGGCATTGTCA-3′
POLR3C	F: 5′-ACTGGTGCAGAGGAAGCACA-3′	R: 5′-TCTAGCTGCTGACGTTCAGGAG-3′
POLR3D	F: 5′-ACCCTGGCTGACCTGACAGA-3′	R: 5′-AGGAGTTGCACCCTTCCAGA-3′
POLR3F	F: 5′-AGAAGGCACAGTTGGCAGTGT-3′	R: 5′-TGGGAGGGATGATTGGATTG-3′
$tRNA_i^{Met}$	F: 5′-AGAGTGGCGCAGCGGAA-3′	R: 5′-TAGCAGAGGATGGTTTCGATCC-3′
$tRNA_e^{Met}$	F: 5′-GCCTCGTTAGCGCAGTAGGTA-3′	R: 5′-GAGGATCGAACTCACGACCTTC-3′
$tRNA^{Pro}$	F: 5′-ATTTGAACCCGGGACCTCTC-3′	R: 5′-GGGTATGATTCTCGCTTAGGGTG-3′
U6 RNA	F: 5′-CTCGCTTCGGCAGCACATATA-3′	R: 5′-AACGCTTCACGAATTTGCG-3′
7SK RNA	F: 5′-TCTTCGGTCAAGGGTATACGAGTAG-3′	R: 5′-CAAATGGACCTTGAGAGCTTGTT-3′
7SL RNA	F: 5′-GCACTAAGTTCGGCATCA-3′	R: 5′-TTTGACCTGCTCCGTTTC-3′
vault1 RNA	F: 5′-GGCTGGCTTTAGCTCAGCG-3′	R: 5′-TCTCGAACAACCCAGACAGGT-3′
BC200 RNA	F: 5′-GGTGGCTCACGCCTGTAATC -3′	R: 5′-GAACTCCTGGGCTCAAGCTATC-3′
5S rRNA	F: 5′-CTGAACGCGCCCGATCT-3′	R: 5′-GCGGTCTCCCATCCAAGTAC-3′
FOXA1	F: 5′-CGGAGCAGCAGCATAAG-3′	R: 5′-GCAACGTAGAGCCGTAAG-3′
RPL13A	F: 5′- GGGAGCAAGGAAAGGGTCTTA-3′	R: 5′-CACCTGCACAATTCTCCGAGT-3′
RPL29	F: 5′-GGCTATCAAGGCCCTCGTAAA-3′	R: 5′-CGAGCTTGCGGCTGACA-3′
